# A framework for research on recurrent acute disasters

**DOI:** 10.1126/sciadv.abk2458

**Published:** 2022-03-09

**Authors:** Gary E. Machlis, Miguel O. Román, Steward T. A. Pickett

**Affiliations:** 1 Clemson University, Clemson, SC, USA.; 2 Universities Space Research Association, Washington, DC, USA.; 3 Cary Institute of Ecosystem Studies, Millbrook, NY, USA.

## Abstract

Disaster science examines the causes, behaviors, and consequences of hazardous events, from hurricanes to wildfires, flooding, and major industrial accidents. Individual disasters are recurring more frequently and with greater intensity. Recurrent acute disasters (RADs) are sequential disasters that affect a specific locale over time. While disaster science has matured in recent years, understanding of the distinctive characteristics of RADs varies by discipline and lacks predictive power. A theoretical framework is presented by borrowing in part from mathematical topology and disturbance ecology. The recurrent disasters affecting Puerto Rico 2017–2020 are examined as a case example to test the framework. A key variable is the complex characteristics of legacy conditions created by one disaster that influence the effects of subsequent disasters. Substantial improvements in disaster response, recovery, and preparedness can be gained by adopting a RAD-based approach.

## Teaser

As disasters become more frequent and intense, research on legacy conditions can improve disaster science and preparedness.

## INTRODUCTION

Individual disasters include hurricanes, wildfires, earthquakes, floods, tornadoes, cyclones, windstorms, landslides, severe droughts, major industrial and technological accidents, and combinations of these events (sometimes described as compound disasters). These individual disasters are recurring more frequently with potentially greater cumulative impact—from repeated extreme heat events in the American West to periodic European flooding and hurricanes in the Caribbean (*1*) .

Recurrent acute disasters (RADs) are sequential disasters that affect a specific locale over time. These disasters have the potential to create conditions that alter the effects of subsequent disasters. For example, Hurricane Irma traversed part of the Greater Antilles in September 2017. The hurricane created conditions such as saturated soils, weakened energy grids, and infrastructure damage that amplified the subsequent effects of Hurricane Maria 2 weeks later (*2*). In California, drought conditions combined with high temperatures and strong winds intensified the Dixie Fire that burned more than 963,000 acres in July 2021. The fire created burn scar conditions that exacerbated water runoff from the subsequent atmospheric river storms of October 2021 and led to major debris flows, mudslides, and property damage (*3*).

Driven in part by climate change, population growth in at-risk locations, and inadequate disaster preparedness, RADs pose an increasing threat to environmental quality, economic activity, public health, and safety. While disaster science has matured in recent years, understanding the distinctive characteristics of RADs varies by discipline, is largely siloed, and lacks predictive power (*4*). Several important research questions emerge. Is there a distinctive signature to RADs and their effects on human ecosystems? If so, what are the key legacy conditions that create distinctive signatures, and can a theory be developed that could predict them? And if so, what policies or planning strategies can be undertaken to mitigate the effects of RADs and support preparedness, disaster management, and community resilience? Here, we construct and initially test a new and novel framework for improving the scientific understanding of RADs, apply the framework to a case example, and describe its policy implications.

### Background

The number of internationally recognized disasters rose from 4212 during 1980–1999 to 7348 during 2000–2019 (*5*). Disasters are also increasing in intensity and frequency. Floods are covering greater areas and recurring at shorter intervals. Tropical cyclones are shifting poleward and increasing in frequency, amounts of rainfall, proximity to land, and storm surge flooding. Wildfires have risen in number, area, severity, and length of fire season. Meteorological heat domes and repeated urban heat extremes have emerged as a public health hazard. Climate change is now recognized as an underlying factor and accelerant in the increase and intensity of these events (*6*, *7*).

The human cost of disasters is also rising. While deaths have remained somewhat constant—from 1980 to 1999, disasters killed 1.19 million persons, and from 2000 to 2019, 1.23 million persons were killed—economic losses rose by 82% (adjusted for inflation) during the same periods from $1.63 trillion to $2.97 trillion (*5*). Evidence suggests that these impacts are differentially distributed, with the most vulnerable populations and communities being those of low income, persons of color, and those with underrepresentation in governance, policy, and recovery planning (*7*, *8*).

As the intensity and frequency of disasters increase, RADs will become more common and the human costs in terms of mortality, health, well-being, environmental quality, and economic security will be exacerbated. A better understanding of RADs is critical to improve preparedness, disaster management, response and recovery, and community resilience.

### A framework for advancing research on RADs

Disaster science has largely focused on individual events. Where disasters have been linked, the primary variable has been the time lag between disasters (*9*). Notable exceptions have been engineering studies of weakened structures (*10*) and the work of historians, where recurrence of disasters over historical periods (primarily hurricanes in the Caribbean) has revealed important ecological and socioeconomic linkages between disasters (*11*, *12*). Theory that would generate sound predictive models is underdeveloped. Disturbance ecology provides a potential foundation (*13*, *14*), as disasters can be defined as disturbances that harm human interests (life, health, property, governance, and so forth). Given the complex coupling of social and environmental systems, a strict dichotomy between “natural” and “human-caused” disasters is neither warranted nor productive.

**Fig. 1.**
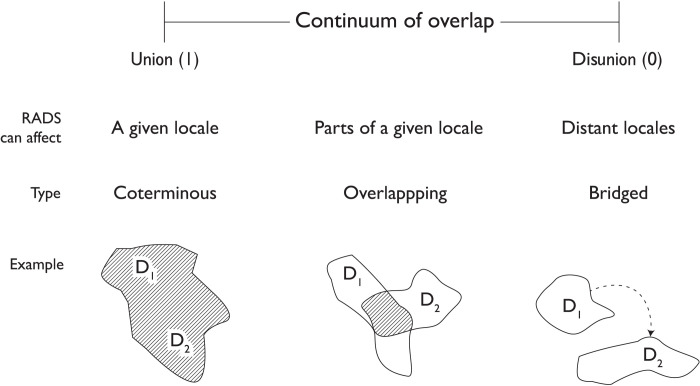
A continuum of RAD spatial relationships. D_1_ and D_2_ represent RADs. Note that complete union (perfect spatial overlap of disasters) is improbable and disunion can vary by the spatial distance between bridged events.

Key concepts can be operationalized to construct a comprehensive framework to advance research on RADs. Borrowing from topology (the mathematical study of geometric properties and spatial relations), the spatial relationship between RADs can be represented by a continuum from complete spatial overlap or union (overlap = 1) to spatially discrete or disunion (overlap = 0) and described as coterminous, overlapping, or bridged—where a disaster in one locale creates specific impacts on a different locale (see Fig. 1). RADs can vary in duration of an event in a specific locale, frequency (the number of RADs over a time frame), periodicity (the strength of cyclical pattern between disasters), and expansion rate (how events develop across the landscape). Disasters are characterized as either previous or subsequent, depending on their chronological sequence.

Each separate disaster has an inventory of effects on a human ecosystem. We follow the precedent of disturbance theory (*13*, *15*) in using the term “effects” to indicate that the outcomes of disaster can be either positive or negative. An example of a positive effect is improved adaptive management strategies that emerged after severe heat waves (*15*, *16*). Effects can be negative when limiting resources are lost as a result of the disaster, such as erosion of topsoil from slopes after a fire (*17*).

The effects of recurrent disasters may also be created through potential cascades of consequences, which can begin with either biophysical components or social effects of the system. For example, severe fire that kills woody plants binding soil makes slopes more susceptible to erosion during subsequent rainstorms, leading to mudslides that destroy infrastructure and buildings and can generate a threat to life and livelihoods. The tight feedbacks between the biophysical and social components of human ecosystems are crucial aspects of the chains of consequences and generation of positive or negative effects of a disaster. In addition, postdisaster consequences can migrate from biological components to social components within tightly coupled human ecosystems (*18*).

**Fig. 2.**
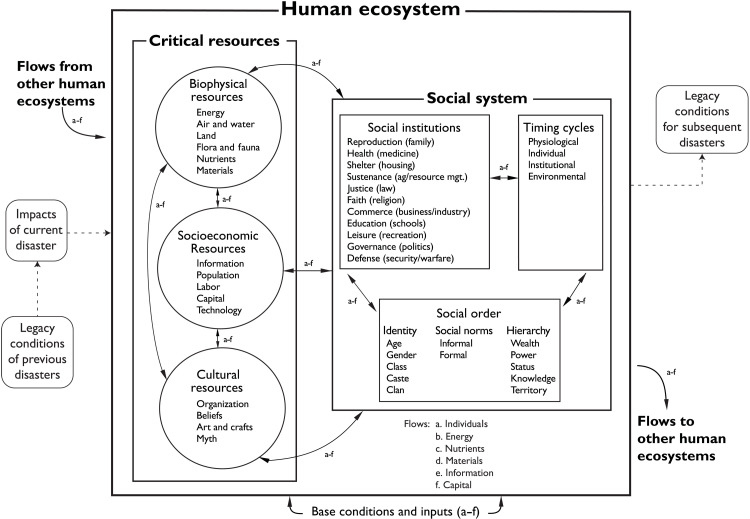
A conceptual model of how RADs affect human ecosystems [adapted from (*18*)]. The arrows represent flows of (a) individuals, (b) energy, (c) nutrients, (d) materials, (e) information, and (f) capital. These flows connect the critical resources and social components of the human ecosystem.

The human ecosystem model used here (see Fig. 2) has been applied to disasters such as the Deepwater Horizon oil spill and Hurricane Sandy (*19*), applications in which its ability to incorporate the interactions between biophysical and social subsystems during and after disaster has been crucial. Human responses to disasters do not occur in a vacuum relative to biophysical responses, and both subsystems have different states and speeds of effects. These effects and chains of consequences create a disaster’s signature. For RADs, there is a distinctive signature—the inventory of cumulative effects and cascades of consequences associated with previous disasters that critically influence subsequent disasters.

**Fig. 3.**
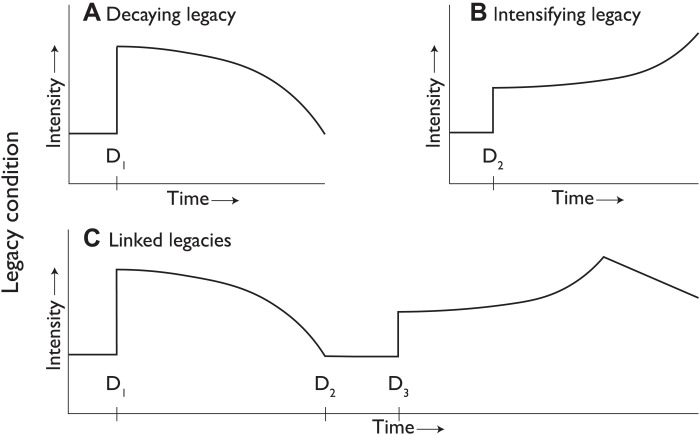
A general schema of several categories of legacy conditions. (**A** to **C**) D_1_ to D_3_ represent RADs.

Postdisaster conditions that persist within the human ecosystem are defined as legacy conditions. Legacy conditions influence the behavior and impacts of subsequent disasters in the same locale. Legacy conditions can have either positive or negative effects. Some legacy conditions can decay over time, and others can intensify. Legacy conditions are the critical link in creating a series of RADs or a RAD set (see Fig. 3).

### An example: Puerto Rico 2017–2020

Located in the Greater Antilles, Puerto Rico has historically experienced the seasonal Atlantic hurricane season as a series of RADs. For example, Lugo (*2*) describes land cover changes created by previous hurricanes San Felipe (1928) and San Ciprián (1932) as “ecological legacies” influencing the effects of subsequent hurricanes. Hence, it provides a useful case in applying the RAD framework.

**Fig. 4.**
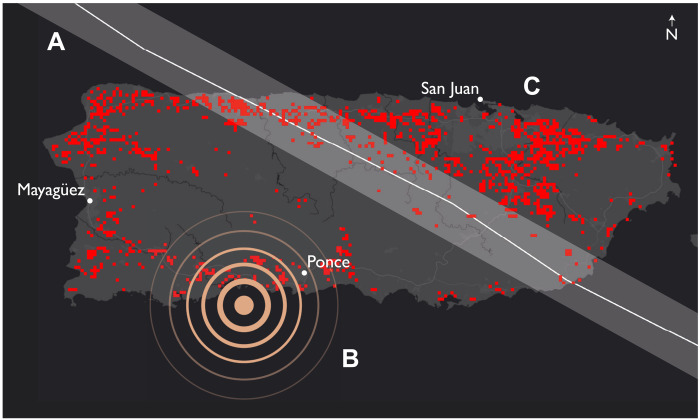
Map of the main island of Puerto Rico showing two bridged RADs. Hurricane Maria (2017) caused major energy infrastructure damage (**A**); the 2020 earthquake series in the south of Puerto Rico (**B**) caused immediate and widespread power outages in the earthquake zone and the already vulnerable northern portion of the island (**C**).

In September 2017, Hurricane Irma passed close to Puerto Rico. Torrential rainfall and high winds led to widespread power outages, saturated ground, a weakened energy grid, and impaired civil infrastructure. Emergency supplies in Puerto Rico were sent to neighboring Caribbean islands that suffered more catastrophic damage. Two weeks later, Hurricane Maria moved directly across Puerto Rico. Partly because of the previous hurricane and the disrupted services, collapsing health system, and devastated infrastructure, Puerto Rico was overwhelmed. Emergency supplies, distribution systems, and recovery responses were inadequate, leading to the deaths of more than 4000 persons (*20*). Then, in 2019, an island-wide drought forced Puerto Rico to ration water. In early 2020, a series of earthquakes (maximum = 6.4 magnitude) affected the archipelago. The fragile water distribution system, housing, and energy infrastructure within the earthquake’s epicenter had already been weakened, damaged, or destroyed; the earthquakes caused substantial additional damage, including long-duration power failures. Figure 4 illustrates the resulting spatial relationship (in this case, bridged) between these previous and subsequent disasters. The assessment of the spatial distribution of earthquake-triggered power outages (Fig. 4, highlighted in red) is based on satellite-derived estimates using NASA’s Black Marble nighttime lights product suite, using the methods described by Román *et al*. (*21*).

RADs such as Hurricane Irma, Hurricane Maria, the island’s drought, and earthquakes have had cumulative and cascading impacts on Puerto Rico’s interconnected human ecological systems. This has led to negative legacy conditions including a deteriorating energy infrastructure (*22*), public loss of trust in government institutions (*23*), and a health care system under immense strain. Positive legacy conditions include Puerto Rico’s heightened community-wide awareness and perceptions of health risks since Hurricane Maria, which may have influenced Puerto Rico’s high coronavirus disease 2019 vaccination rate (73%) and public adherence to pandemic containment measures (*24*, *25*).

### Advancing RAD research

This framework applied to the Puerto Rico case can provide a useful understanding and operationalized measures for examining other RADs and their effects. We can propose that the effects of a subsequent acute disaster on the human ecosystem at *T*_1_ are a function of:

1) the condition of the human ecosystem at *T*_0_;
2) the legacy conditions of previous disaster(s);
3) the geospatial union (0 to 1) of previous disaster(s) and the subsequent disaster;
4) the time gap between previous and subsequent disasters;
5) the response, recovery, and preparedness actions taken during the time gap; and
6) the type, intensity, and expansion rate of the subsequent disaster.

An increasingly rich collection of data sources and online tools are available to test RAD theory, including satellite-derived data, improved modeling approaches (e.g., seismic, landslide, and tropical cyclone risk and susceptibility mapping), field-collected data (e.g., demographic, anthropological, and epidemiological surveys), mobility tracking, and social media data. Legacy conditions of RADs can be characterized through multihazard and locally informed data collection strategies that capture and document persistent conditions and include impacts on vulnerable and underrepresented populations. For example, recent studies have used electricity restoration as proxy for more general postdisaster recovery following events like Hurricane Maria and the Puerto Rico earthquakes (*22*). Extended periods without access to electricity affect how long communities may go without basic needs, such as water and food, transportation, and medical assistance. These conditions also increase reliance on individual responsibility and social networks while heightening vulnerability to homelessness and economic insecurity—creating legacy conditions that challenge conventional wisdom about postdisaster recovery timelines (*26*).

## DISCUSSION

The framework described above has considerable potential to advance disaster science as it strives to account for increasing RADs. The policy implications of a robust understanding of RADs and its application to specific locales are substantial.

As legacy conditions are critical factors affecting future disasters, response and recovery actions should include immediate and detailed monitoring of key conditions and the development of legacy scenarios for subsequent disasters. Understanding legacy conditions can also alert emergency managers to hidden threats and response needs. Because legacy conditions have differential impacts on vulnerable communities, issues of environmental justice can be better addressed by RAD-sensitive disaster and recovery policies.

To reduce risk from future disasters, preparedness programs should update incident response plans to include specific consideration of how previous disasters have altered the response landscape and available resources. RAD-focused disaster science can provide essential inputs of spatial and temporal data as well as improved predictive theory and analysis tools. In addition, increased attention to building codes, public health regulations, private insurance premiums, emergency communications, prepositioning of resources, and community preparedness training can all benefit from a better understanding of RADs.

The Puerto Rico case illustrates these generalized policy implications. A key sector that stands to benefit from RAD-sensitive response and recovery actions is the housing sector. Approximately 18% of occupied housing units in the Municipality of Guánica (the 2019 earthquake’s primary epicenter) had already been severely damaged or destroyed 2 years prior by Hurricane Maria (*27*). Immediate and detailed monitoring of key conditions such as the total number and location of damaged or vulnerable housing units and the type and severity of damage would have enabled the development of legacy scenarios and preparedness plans for subsequent disasters like Puerto Rico’s 2019 earthquakes.

Understanding legacy conditions can also alert recovery crews about preexisting infrastructure and social vulnerabilities. After Hurricane Maria, authorities installed 1601 portable electricity generators—the largest disaster generator mission in U.S. history (*28*). Data on their distribution and use combined with near real-time assessments of electricity restoration efforts (*22*) could enable future incident response plans with specific information about vulnerable communities and households that have traditionally shouldered the longest outages.

Damage and needs assessments should also systematically include legacy conditions as a consideration in priorities, design, and implementation. One example is the development of policies that foster land conservation and the protection of critical natural resources to maximize land and water resources for agriculture and urban uses (*2*). The El Yunque National Forest provides approximately 50% of the water supply for the San Juan metro area. The drainage basin also provides water to various municipalities located downstream from the national forest. If the periodicity of RAD-like events changes from every 50 years to shorter intervals (e.g., 10 years), then the forest ecosystem may not have enough time to recover. Specific research and development efforts to understand how tropical forests around the world will fare in a RAD-like future are essential. These efforts could also lead to improved understanding of how critical ecosystem functions and services will be able to mediate the effects of other stressors such as the El Niño/La Niña–Southern Oscillation on drought behavior across Puerto Rico during the May to November wet season (*29*).

Research on RADs is clearly warranted, and the framework presented here provides one path forward. Of particular importance is testing the framework with other disaster sets (such as recurrent tornadoes in the American Midwest or repeated extreme events in European cities) and improving the ability to monitor and identify legacy conditions soon after an individual disaster. For the disaster management community, it will be necessary to convert the RAD-based predictions of legacy conditions into specific preparedness actions that reduce vulnerability to subsequent disasters. For policy-makers to adopt more RAD-based approaches, it will be imperative to document their relative advantages, application to specific locales, trialability, and other characteristics of successful innovations. Increasing our understanding of RADs has the potential to advance disaster science, improve disaster response, build resilience to future disasters, and save lives.
